# (Methanol-κ*O*)bis­{2-meth­oxy-6-[(4-methyl­phen­yl)iminiometh­yl]phenolato-κ^2^ *O*,*O*′}tris­(nitrato-κ^2^ *O*,*O*′)lanthanum(III)

**DOI:** 10.1107/S1600536810038109

**Published:** 2010-09-30

**Authors:** Jia-Lu Liu, Hai-Ting Cai, Guo-Liang Zhao

**Affiliations:** a College of Chemistry and Life Sciences and Xingzhi College, Zhejiang Normal University, Jinhua 321004, People’s Republic of China

## Abstract

The asymmetric unit of title compound, [La(NO_3_)_3_(C_15_H_15_NO_2_)_2_(CH_3_OH)], consists of two Schiff base 2-meth­oxy-6-[(4-methyl­phen­yl)iminiometh­yl]phenolato (H*L*) ligands, three independent nitrate anions and one methanol mol­ecule coordinated to La^III^. The coordination environment of the La^III^ ion is formed by eleven O atoms. Three bidentate nitrate anions coordinate to the La^III^ ion, while two H*L* ligands chelate the metal center with O atoms from the phenolate and meth­oxy groups. The H*L* ligands are zwitterionic, with protonated imine N atoms. The coordination sphere is completed by one methanol mol­ecule. The protonated imine N atoms are involved in intra­molecular N—H⋯O hydrogen bonds with the phen­oxy groups and nitrate ligands. One O atom of one nitrate group is disordered over two sites of equal occupancy.

## Related literature

For Schiff base ligands derived from *o*-vanillin and aniline and their rare earth complexes, see: Burrows & Bailar (1966 ▶); Li *et al.* (2008 ▶); Xian *et al.* (2008 ▶); Zhao *et al.* (2007 ▶). For their applications, see: Leadbeater & Marco (2002 ▶); Quici *et al.* (2004 ▶).
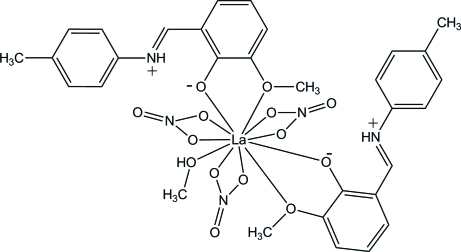


## Experimental

### 

#### Crystal data

[La(NO_3_)_3_(C_15_H_15_NO_2_)_2_(CH_4_O)]
*M* *_r_* = 839.54
Triclinic, 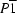

*a* = 7.8616 (2) Å
*b* = 14.6502 (5) Å
*c* = 16.6470 (5) Å
α = 73.210 (2)°
β = 85.648 (2)°
γ = 79.320 (1)°
*V* = 1803.26 (9) Å^3^
*Z* = 2
Mo *K*α radiation
μ = 1.26 mm^−1^
*T* = 296 K
0.43 × 0.31 × 0.20 mm

#### Data collection

Bruker APEXII CCD area-detector diffractometer
Absorption correction: multi-scan (*SADABS*; Sheldrick, 1996 ▶) *T*_min_ = 0.634, *T*_max_ = 0.783
30011 measured reflections
8464 independent reflections
7465 reflections with *I* > 2σ(*I*)
*R*_int_ = 0.024

#### Refinement

*R*[*F* ^2^ > 2σ(*F* ^2^)] = 0.034
*wR*(*F* ^2^) = 0.101
*S* = 1.08
8464 reflections
473 parameters
H atoms treated by a mixture of independent and constrained refinement
Δρ_max_ = 1.23 e Å^−3^
Δρ_min_ = −0.51 e Å^−3^

### 

Data collection: *APEX2* (Bruker, 2006 ▶); cell refinement: *SAINT* (Bruker, 2006 ▶); data reduction: *SAINT*; program(s) used to solve structure: *SHELXS97* (Sheldrick, 2008 ▶); program(s) used to refine structure: *SHELXL97* (Sheldrick, 2008 ▶); molecular graphics: *SHELXTL* (Sheldrick, 2008 ▶); software used to prepare material for publication: *SHELXTL*.

## Supplementary Material

Crystal structure: contains datablocks I, global. DOI: 10.1107/S1600536810038109/bh2313sup1.cif

Structure factors: contains datablocks I. DOI: 10.1107/S1600536810038109/bh2313Isup2.hkl

Additional supplementary materials:  crystallographic information; 3D view; checkCIF report

## Figures and Tables

**Table 1:** Hydrogen-bond geometry (Å, °)

*D*—H⋯*A*	*D*—H	H⋯*A*	*D*⋯*A*	*D*—H⋯*A*
N1—H1*A*⋯O1	0.86	2.01	2.668 (3)	132
N1—H1*A*⋯O11	0.86	2.52	3.307 (4)	153
N2—H2*A*⋯O3	0.86	1.95	2.627 (3)	135
N2—H2*A*⋯O6	0.86	2.65	3.440 (5)	154

**Fig. 1.**
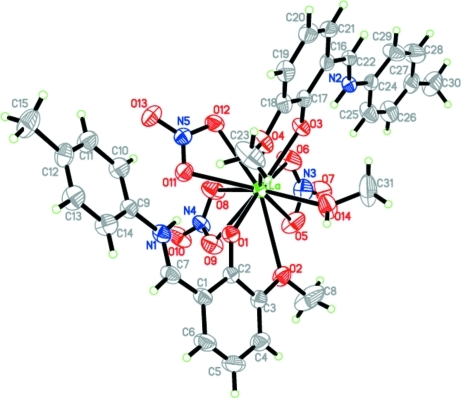
The molecular structure of the title complex, showing the atom-labelling scheme. Displacement ellipsoids are drawn at the 30% probability level.

## supplementary crystallographic information

### Comment

Schiff base ligands derived from substituted *o*-vanillin and aniline and their rare earth metal complexes have generated considerable attention in the past decades, due to their intriguing novel structural features (Burrows & Bailar, 1966; Zhao *et al.*, 2007; Xian *et al.*, 2008; Li *et al.*, 2008) and promising applications in various fields such as catalysis, optoelectronic devices, and so on (Leadbeater & Marco, 2002; Quici *et al.*, 2004). Interested in this field, we have been engaged in a major effort directed toward the synthesis of new analogous Schiff base derived from *o*-vanillin and their rare metal complexes. In few previous articles, we have reported our partial research results (Zhao *et al.*, 2007; Xian *et al.*, 2008; Li *et al.*, 2008). Herein, we describe a new La(III) complex.

The single-crystal structure is shown in Fig. 1, which illustrates that the La(III) ion in this complex is eleven-coordinated by six O atoms from three nitrate radical ions, four O atoms from the Schiff bases, and one O atom from methanol. The Schiff bases are coordinated to the La(III) ion in the bidentate mode, using O atoms from methoxy groups and deprotonated phenolic hydroxyl groups. The bonds between La(III) and O atoms from phenoxy groups are 2.429 (2) and 2.482 (2) Å, which are shorter than those between La(III) and O atoms of methoxy groups [2.808 (2) and 2.964 (3) Å]. The nitrate radical anions coordinate to the La(III) with O atoms with the distances ranging from 2.574 (19) to 2.741 (3) Å, which are intermediate between the La—O(phenolic) and the La—O(methoxy) bond lengths. The La—O(methanol) bong length is only slightly longer than the La—O(phenolic). In addition, the O5 atom in a nitrate anion is disordered over two sites.

The hydrogen bonds and π···π weak non-covalent interactions give stability to the crystal structure. In each H*L* ligand, the proton of phenolic hydroxyl group is transferred to the imine N atom, which is involved in the formation of intramolecular hydrogen bonds. There are no classic hydrogen bonds between the adjacent molecules. π···π interactions exist in the crystal between symmetry-related molecules.

### Experimental

Reagents and solvents are commercially available and were not purified before use. The Schiff base ligand 2-[(4-methylphenyl)iminomethyl]-6-methoxy-phenol was synthesized by condensation of *o*-vanillin and *p*-methylaniline. The title complex was obtained by adding La(NO_3_)_3_ (1 mmol, dissolved in 20 ml methanol) to *N*-salicylidene-*p*-toluidine (2 mmol) in methanol. The mixture was stirred for 8 h at room temperature. The resulting solid was then filtered out and the reddish-brown solution was kept aside. Red crystals were obtained after several days.

### Refinement

The structure was solved by direct methods and successive Fourier difference synthesis. The H atoms bonded to C and N atoms were positioned geometrically and refined using a riding model [aliphatic C—H =0.96 Å (*U*_iso_(H) = 1.5*U*_eq_(C)), aromatic C—H = 0.93 Å (*U*_iso_(H) = 1.2*U*_eq_(C)) and N—H = 0.86 Å, *U*_iso_(H) = 1.2*U*_eq_(N)]. H atom of the hydroxyl group in methanol, H14, was found in a difference map and refined freely.

### Crystal data

**Table:**

[La(NO_3_)_3_(C_15_H_15_NO_2_)_2_(CH_4_O)]	*Z* = 2
*M**_r_* = 839.54	*F*(000) = 848
Triclinic, *P*1	*D*_x_ = 1.546 Mg m^−^^3^
Hall symbol: -P 1	Mo *K*α radiation, λ = 0.71073 Å
*a* = 7.8616 (2) Å	Cell parameters from 9937 reflections
*b* = 14.6502 (5) Å	θ = 1.5–27.7°
*c* = 16.6470 (5) Å	µ = 1.26 mm^−^^1^
α = 73.210 (2)°	*T* = 296 K
β = 85.648 (2)°	Block, red
γ = 79.320 (1)°	0.43 × 0.31 × 0.20 mm
*V* = 1803.26 (9) Å^3^	

### Data collection

**Table:**

Bruker APEXII CCD area-detector diffractometer	8464 independent reflections
Radiation source: fine-focus sealed tube	7465 reflections with *I* > 2σ(*I*)
graphite	*R*_int_ = 0.024
φ and ω scans	θ_max_ = 27.7°, θ_min_ = 1.5°
Absorption correction: multi-scan (*SADABS*; Sheldrick, 1996)	*h* = −9→10
*T*_min_ = 0.634, *T*_max_ = 0.783	*k* = −19→18
30011 measured reflections	*l* = −21→21

### Refinement

**Table:**

Refinement on *F*^2^	Primary atom site location: structure-invariant direct methods
Least-squares matrix: full	Secondary atom site location: difference Fourier map
*R*[*F*^2^ > 2σ(*F*^2^)] = 0.034	Hydrogen site location: inferred from neighbouring sites
*wR*(*F*^2^) = 0.101	H atoms treated by a mixture of independent and constrained refinement
*S* = 1.08	*w* = 1/[σ^2^(*F*_o_^2^) + (0.0579*P*)^2^ + 0.8812*P*] where *P* = (*F*_o_^2^ + 2*F*_c_^2^)/3
8464 reflections	(Δ/σ)_max_ = 0.001
473 parameters	Δρ_max_ = 1.23 e Å^−^^3^
0 restraints	Δρ_min_ = −0.51 e Å^−^^3^
0 constraints	

### Fractional atomic coordinates and isotropic or equivalent isotropic displacement parameters (Å^2^)

**Table:**

	*x*	*y*	*z*	*U*_iso_*/*U*_eq_	Occ. (<1)
La	0.50959 (2)	0.172767 (11)	0.194416 (10)	0.04293 (7)	
O1	0.3524 (3)	0.32578 (16)	0.11163 (14)	0.0509 (5)	
O2	0.4089 (4)	0.20682 (18)	0.01876 (17)	0.0673 (7)	
O3	0.4311 (3)	0.05673 (16)	0.32705 (14)	0.0534 (5)	
O4	0.2435 (3)	0.23100 (17)	0.30077 (18)	0.0668 (7)	
O5	0.665 (2)	0.0504 (12)	0.1156 (11)	0.093 (5)	0.50
O5'	0.610 (3)	0.0211 (12)	0.1433 (13)	0.118 (7)	0.50
O6	0.7262 (5)	−0.0014 (2)	0.2482 (2)	0.0983 (11)	
O7	0.8172 (5)	−0.0934 (3)	0.1712 (3)	0.1174 (14)	
O8	0.8476 (4)	0.1764 (3)	0.17935 (19)	0.0857 (9)	
O9	0.7128 (4)	0.2593 (2)	0.06973 (19)	0.0807 (9)	
O10	0.9881 (4)	0.2497 (3)	0.0703 (2)	0.0941 (11)	
O11	0.5728 (4)	0.32810 (17)	0.22884 (17)	0.0652 (6)	
O12	0.6419 (4)	0.19922 (19)	0.32788 (15)	0.0659 (6)	
O13	0.6879 (4)	0.3357 (2)	0.34005 (19)	0.0815 (8)	
O14	0.2457 (4)	0.1086 (3)	0.1706 (2)	0.0912 (11)	
N1	0.2882 (4)	0.50830 (19)	0.11960 (17)	0.0521 (6)	
H1A	0.3398	0.4502	0.1421	0.063*	
N2	0.5013 (3)	−0.12440 (19)	0.41794 (17)	0.0486 (6)	
H2A	0.5240	−0.0801	0.3738	0.058*	
N3	0.7281 (5)	−0.0207 (2)	0.1827 (3)	0.0753 (9)	
N4	0.8512 (4)	0.2286 (2)	0.10540 (19)	0.0582 (7)	
N5	0.6358 (4)	0.2885 (2)	0.29964 (18)	0.0542 (6)	
C1	0.2592 (5)	0.4606 (2)	−0.0041 (2)	0.0548 (8)	
C2	0.3205 (4)	0.3609 (2)	0.03234 (19)	0.0453 (6)	
C3	0.3459 (4)	0.3002 (2)	−0.0216 (2)	0.0523 (7)	
C4	0.3071 (6)	0.3360 (3)	−0.1045 (2)	0.0694 (10)	
H4A	0.3238	0.2945	−0.1386	0.083*	
C5	0.2422 (7)	0.4346 (3)	−0.1392 (2)	0.0822 (13)	
H5A	0.2164	0.4580	−0.1959	0.099*	
C6	0.2170 (7)	0.4957 (3)	−0.0904 (2)	0.0775 (12)	
H6A	0.1720	0.5608	−0.1133	0.093*	
C7	0.2388 (5)	0.5277 (2)	0.0433 (2)	0.0611 (9)	
H7A	0.1858	0.5907	0.0177	0.073*	
C8	0.4344 (12)	0.1407 (4)	−0.0311 (5)	0.162 (4)	
H8C	0.4955	0.0794	0.0006	0.194*	
H8A	0.5006	0.1657	−0.0808	0.194*	
H8B	0.3240	0.1327	−0.0465	0.194*	
C9	0.2656 (4)	0.5737 (2)	0.1702 (2)	0.0512 (7)	
C10	0.3521 (6)	0.5460 (3)	0.2447 (3)	0.0675 (10)	
H10A	0.4255	0.4866	0.2605	0.081*	
C11	0.3294 (6)	0.6071 (3)	0.2957 (3)	0.0724 (11)	
H11A	0.3875	0.5882	0.3460	0.087*	
C12	0.2219 (5)	0.6955 (3)	0.2731 (3)	0.0651 (9)	
C13	0.1410 (6)	0.7226 (3)	0.1976 (3)	0.0747 (11)	
H13A	0.0713	0.7832	0.1808	0.090*	
C14	0.1595 (6)	0.6631 (3)	0.1459 (3)	0.0683 (10)	
H14A	0.1018	0.6826	0.0954	0.082*	
C15	0.1923 (7)	0.7602 (4)	0.3311 (4)	0.0954 (16)	
H15A	0.1148	0.8187	0.3055	0.143*	
H15B	0.3008	0.7758	0.3409	0.143*	
H15C	0.1427	0.7272	0.3834	0.143*	
C16	0.3056 (4)	−0.0007 (2)	0.4620 (2)	0.0464 (7)	
C17	0.3251 (4)	0.0727 (2)	0.3862 (2)	0.0463 (6)	
C18	0.2207 (4)	0.1652 (2)	0.3780 (2)	0.0535 (7)	
C19	0.1127 (5)	0.1833 (3)	0.4407 (3)	0.0617 (9)	
H19A	0.0462	0.2444	0.4338	0.074*	
C20	0.0999 (5)	0.1110 (3)	0.5159 (3)	0.0654 (10)	
H20A	0.0266	0.1249	0.5589	0.078*	
C21	0.1928 (5)	0.0211 (3)	0.5269 (2)	0.0574 (8)	
H21A	0.1826	−0.0264	0.5771	0.069*	
C22	0.3935 (4)	−0.0961 (2)	0.4727 (2)	0.0495 (7)	
H22A	0.3732	−0.1422	0.5225	0.059*	
C23	0.1142 (7)	0.3155 (3)	0.2773 (4)	0.108 (2)	
H23A	0.0386	0.3197	0.3247	0.162*	
H23B	0.0483	0.3120	0.2324	0.162*	
H23C	0.1687	0.3718	0.2591	0.162*	
C24	0.5857 (4)	−0.2201 (2)	0.4228 (2)	0.0514 (7)	
C25	0.6855 (6)	−0.2367 (3)	0.3564 (3)	0.0787 (12)	
H25A	0.6994	−0.1853	0.3096	0.094*	
C26	0.7662 (6)	−0.3292 (3)	0.3578 (3)	0.0841 (13)	
H26A	0.8345	−0.3387	0.3118	0.101*	
C27	0.7489 (6)	−0.4063 (3)	0.4239 (3)	0.0730 (11)	
C28	0.6463 (9)	−0.3883 (3)	0.4903 (3)	0.1021 (18)	
H28A	0.6307	−0.4400	0.5365	0.122*	
C29	0.5661 (8)	−0.2968 (3)	0.4906 (3)	0.0885 (15)	
H29A	0.4988	−0.2871	0.5368	0.106*	
C30	0.8370 (9)	−0.5076 (4)	0.4238 (4)	0.109 (2)	
H30A	0.8078	−0.5530	0.4750	0.163*	
H30B	0.9602	−0.5103	0.4195	0.163*	
H30C	0.7988	−0.5234	0.3769	0.163*	
C31	0.1924 (9)	0.0164 (4)	0.2049 (5)	0.137 (3)	
H31A	0.0837	0.0174	0.1818	0.205*	
H31B	0.1798	0.0030	0.2648	0.205*	
H31C	0.2781	−0.0330	0.1912	0.205*	
H14	0.166 (8)	0.151 (4)	0.140 (4)	0.107 (18)*	

### Atomic displacement parameters (Å^2^)

**Table:**

	*U*^11^	*U*^22^	*U*^33^	*U*^12^	*U*^13^	*U*^23^
La	0.04001 (10)	0.03162 (10)	0.05135 (11)	−0.00059 (6)	−0.00069 (7)	−0.00613 (7)
O1	0.0568 (13)	0.0394 (11)	0.0507 (12)	0.0028 (9)	−0.0089 (10)	−0.0083 (9)
O2	0.0818 (18)	0.0450 (13)	0.0776 (17)	0.0009 (12)	−0.0210 (14)	−0.0238 (12)
O3	0.0528 (12)	0.0415 (11)	0.0574 (13)	−0.0024 (9)	0.0127 (10)	−0.0076 (10)
O4	0.0578 (14)	0.0412 (12)	0.0850 (17)	0.0052 (10)	0.0178 (13)	−0.0056 (12)
O5	0.110 (12)	0.067 (7)	0.104 (8)	0.032 (6)	−0.050 (7)	−0.043 (6)
O5'	0.101 (11)	0.094 (12)	0.174 (18)	0.049 (8)	−0.058 (10)	−0.087 (12)
O6	0.119 (3)	0.071 (2)	0.086 (2)	0.0160 (19)	0.017 (2)	−0.0171 (17)
O7	0.124 (3)	0.079 (2)	0.137 (3)	0.052 (2)	−0.015 (3)	−0.052 (2)
O8	0.0601 (16)	0.105 (2)	0.0719 (18)	−0.0099 (16)	−0.0042 (14)	0.0051 (17)
O9	0.0552 (16)	0.095 (2)	0.0764 (18)	−0.0136 (15)	−0.0031 (13)	0.0012 (16)
O10	0.0467 (14)	0.114 (3)	0.093 (2)	−0.0199 (16)	0.0056 (14)	0.0177 (19)
O11	0.0798 (17)	0.0453 (13)	0.0675 (15)	−0.0025 (12)	−0.0209 (13)	−0.0115 (11)
O12	0.0837 (18)	0.0540 (15)	0.0525 (13)	−0.0075 (13)	−0.0064 (12)	−0.0044 (11)
O13	0.083 (2)	0.096 (2)	0.0820 (19)	−0.0218 (17)	−0.0105 (15)	−0.0456 (17)
O14	0.0644 (18)	0.075 (2)	0.118 (3)	−0.0223 (16)	−0.0334 (18)	0.0124 (19)
N1	0.0589 (16)	0.0360 (13)	0.0546 (15)	0.0036 (11)	0.0003 (12)	−0.0096 (11)
N2	0.0531 (15)	0.0417 (14)	0.0496 (14)	−0.0111 (11)	−0.0007 (11)	−0.0091 (11)
N3	0.067 (2)	0.0517 (18)	0.103 (3)	0.0121 (16)	−0.003 (2)	−0.0288 (19)
N4	0.0460 (15)	0.0540 (17)	0.0614 (17)	−0.0079 (12)	−0.0002 (13)	0.0039 (13)
N5	0.0507 (15)	0.0585 (17)	0.0544 (15)	−0.0073 (13)	0.0012 (12)	−0.0192 (13)
C1	0.065 (2)	0.0433 (17)	0.0476 (16)	−0.0017 (15)	0.0010 (15)	−0.0053 (13)
C2	0.0413 (15)	0.0434 (16)	0.0487 (16)	−0.0046 (12)	−0.0021 (12)	−0.0103 (13)
C3	0.0495 (17)	0.0488 (18)	0.0593 (18)	−0.0070 (14)	−0.0040 (14)	−0.0166 (15)
C4	0.084 (3)	0.069 (3)	0.060 (2)	−0.011 (2)	−0.0033 (19)	−0.0273 (19)
C5	0.121 (4)	0.071 (3)	0.0464 (19)	−0.008 (3)	−0.010 (2)	−0.0080 (18)
C6	0.114 (4)	0.051 (2)	0.055 (2)	0.000 (2)	−0.009 (2)	−0.0021 (17)
C7	0.079 (2)	0.0389 (17)	0.0545 (19)	0.0021 (16)	0.0017 (17)	−0.0045 (14)
C8	0.268 (10)	0.070 (3)	0.159 (6)	0.047 (5)	−0.110 (7)	−0.072 (4)
C9	0.0521 (17)	0.0398 (16)	0.0586 (18)	−0.0004 (13)	0.0020 (14)	−0.0145 (14)
C10	0.080 (3)	0.0431 (18)	0.073 (2)	0.0112 (17)	−0.016 (2)	−0.0168 (17)
C11	0.086 (3)	0.057 (2)	0.075 (2)	0.002 (2)	−0.020 (2)	−0.0235 (19)
C12	0.061 (2)	0.054 (2)	0.086 (3)	−0.0032 (17)	−0.0025 (19)	−0.0319 (19)
C13	0.078 (3)	0.051 (2)	0.091 (3)	0.0182 (19)	−0.013 (2)	−0.029 (2)
C14	0.075 (2)	0.051 (2)	0.071 (2)	0.0181 (18)	−0.0130 (19)	−0.0204 (17)
C15	0.103 (4)	0.078 (3)	0.121 (4)	0.002 (3)	−0.016 (3)	−0.060 (3)
C16	0.0428 (15)	0.0488 (17)	0.0517 (16)	−0.0143 (13)	−0.0006 (12)	−0.0164 (13)
C17	0.0391 (14)	0.0462 (16)	0.0559 (17)	−0.0101 (12)	0.0028 (12)	−0.0170 (14)
C18	0.0452 (16)	0.0453 (17)	0.069 (2)	−0.0086 (13)	0.0073 (14)	−0.0165 (15)
C19	0.0544 (19)	0.056 (2)	0.079 (2)	−0.0056 (16)	0.0100 (17)	−0.0302 (18)
C20	0.059 (2)	0.081 (3)	0.067 (2)	−0.0169 (19)	0.0161 (17)	−0.040 (2)
C21	0.059 (2)	0.068 (2)	0.0501 (17)	−0.0175 (17)	0.0057 (15)	−0.0211 (16)
C22	0.0530 (17)	0.0502 (18)	0.0459 (15)	−0.0155 (14)	−0.0042 (13)	−0.0098 (13)
C23	0.085 (3)	0.068 (3)	0.124 (4)	0.026 (2)	0.030 (3)	0.013 (3)
C24	0.0543 (18)	0.0441 (17)	0.0582 (18)	−0.0107 (14)	−0.0079 (14)	−0.0149 (14)
C25	0.091 (3)	0.050 (2)	0.088 (3)	−0.011 (2)	0.025 (2)	−0.016 (2)
C26	0.087 (3)	0.065 (3)	0.104 (3)	−0.010 (2)	0.022 (3)	−0.037 (3)
C27	0.080 (3)	0.048 (2)	0.096 (3)	−0.0049 (19)	−0.019 (2)	−0.026 (2)
C28	0.160 (6)	0.052 (2)	0.082 (3)	−0.003 (3)	−0.004 (3)	−0.009 (2)
C29	0.136 (4)	0.053 (2)	0.061 (2)	0.000 (2)	0.014 (3)	−0.0070 (19)
C30	0.128 (5)	0.064 (3)	0.145 (5)	0.008 (3)	−0.027 (4)	−0.054 (3)
C31	0.117 (5)	0.084 (4)	0.205 (8)	−0.049 (4)	−0.060 (5)	0.001 (4)

### Geometric parameters (Å, °)

**Table:**

La—O1	2.429 (2)	C8—H8A	0.9600
La—O3	2.482 (2)	C8—H8B	0.9600
La—O14	2.532 (3)	C9—C10	1.378 (5)
La—O5'	2.574 (19)	C9—C14	1.384 (5)
La—O5	2.59 (2)	C10—C11	1.383 (5)
La—O11	2.640 (2)	C10—H10A	0.9300
La—O8	2.659 (3)	C11—C12	1.376 (5)
La—O12	2.677 (3)	C11—H11A	0.9300
La—O9	2.682 (3)	C12—C13	1.372 (6)
La—O6	2.741 (3)	C12—C15	1.515 (5)
La—O4	2.808 (2)	C13—C14	1.374 (5)
La—O2	2.964 (3)	C13—H13A	0.9300
O1—C2	1.296 (4)	C14—H14A	0.9300
O2—C3	1.355 (4)	C15—H15A	0.9600
O2—C8	1.426 (5)	C15—H15B	0.9600
O3—C17	1.287 (4)	C15—H15C	0.9600
O4—C18	1.388 (4)	C16—C22	1.406 (5)
O4—C23	1.426 (5)	C16—C21	1.416 (5)
O5—N3	1.342 (18)	C16—C17	1.422 (5)
O5'—N3	1.15 (2)	C17—C18	1.423 (4)
O6—N3	1.201 (5)	C18—C19	1.351 (5)
O7—N3	1.218 (4)	C19—C20	1.397 (6)
O8—N4	1.249 (4)	C19—H19A	0.9300
O9—N4	1.225 (4)	C20—C21	1.350 (6)
O10—N4	1.232 (4)	C20—H20A	0.9300
O11—N5	1.248 (4)	C21—H21A	0.9300
O12—N5	1.248 (4)	C22—H22A	0.9300
O13—N5	1.230 (4)	C23—H23A	0.9600
O14—C31	1.434 (6)	C23—H23B	0.9600
O14—H14	0.87 (6)	C23—H23C	0.9600
N1—C7	1.293 (4)	C24—C25	1.361 (5)
N1—C9	1.428 (4)	C24—C29	1.366 (5)
N1—H1A	0.8600	C25—C26	1.380 (6)
N2—C22	1.301 (4)	C25—H25A	0.9300
N2—C24	1.417 (4)	C26—C27	1.350 (7)
N2—H2A	0.8600	C26—H26A	0.9300
C1—C7	1.408 (5)	C27—C28	1.375 (7)
C1—C2	1.416 (4)	C27—C30	1.518 (6)
C1—C6	1.421 (5)	C28—C29	1.373 (6)
C2—C3	1.416 (4)	C28—H28A	0.9300
C3—C4	1.364 (5)	C29—H29A	0.9300
C4—C5	1.403 (6)	C30—H30A	0.9600
C4—H4A	0.9300	C30—H30B	0.9600
C5—C6	1.354 (6)	C30—H30C	0.9600
C5—H5A	0.9300	C31—H31A	0.9600
C6—H6A	0.9300	C31—H31B	0.9600
C7—H7A	0.9300	C31—H31C	0.9600
C8—H8C	0.9600		
			
O1—La—O3	131.51 (8)	O1—C2—C3	120.9 (3)
O1—La—O14	83.19 (10)	O1—C2—C1	121.9 (3)
O3—La—O14	71.17 (10)	C3—C2—C1	117.2 (3)
O1—La—O5'	125.4 (5)	O2—C3—C4	126.2 (3)
O3—La—O5'	84.7 (4)	O2—C3—C2	112.6 (3)
O14—La—O5'	71.2 (4)	C4—C3—C2	121.2 (3)
O1—La—O5	117.8 (4)	C3—C4—C5	120.8 (4)
O3—La—O5	99.1 (3)	C3—C4—H4A	119.6
O14—La—O5	82.3 (4)	C5—C4—H4A	119.6
O5'—La—O5	15.8 (6)	C6—C5—C4	120.2 (4)
O1—La—O11	64.68 (8)	C6—C5—H5A	119.9
O3—La—O11	108.70 (8)	C4—C5—H5A	119.9
O14—La—O11	136.91 (11)	C5—C6—C1	120.1 (4)
O5'—La—O11	151.1 (4)	C5—C6—H6A	119.9
O5—La—O11	137.2 (4)	C1—C6—H6A	119.9
O1—La—O8	109.57 (9)	N1—C7—C1	125.0 (3)
O3—La—O8	112.38 (8)	N1—C7—H7A	117.5
O14—La—O8	151.69 (14)	C1—C7—H7A	117.5
O5'—La—O8	81.0 (4)	O2—C8—H8C	109.5
O5—La—O8	69.4 (4)	O2—C8—H8A	109.5
O11—La—O8	70.22 (10)	H8C—C8—H8A	109.5
O1—La—O12	109.48 (8)	O2—C8—H8B	109.5
O3—La—O12	68.27 (8)	H8C—C8—H8B	109.5
O14—La—O12	134.66 (11)	H8A—C8—H8B	109.5
O5'—La—O12	123.0 (5)	C10—C9—C14	120.1 (3)
O5—La—O12	123.1 (4)	C10—C9—N1	118.6 (3)
O11—La—O12	47.22 (8)	C14—C9—N1	121.4 (3)
O8—La—O12	66.28 (10)	C9—C10—C11	119.7 (3)
O1—La—O9	69.51 (9)	C9—C10—H10A	120.2
O3—La—O9	158.15 (9)	C11—C10—H10A	120.2
O14—La—O9	123.52 (11)	C12—C11—C10	121.0 (4)
O5'—La—O9	85.6 (4)	C12—C11—H11A	119.5
O5—La—O9	69.8 (3)	C10—C11—H11A	119.5
O11—La—O9	72.62 (10)	C13—C12—C11	118.3 (3)
O8—La—O9	46.59 (9)	C13—C12—C15	121.2 (4)
O12—La—O9	101.36 (9)	C11—C12—C15	120.5 (4)
O1—La—O6	165.10 (9)	C12—C13—C14	122.1 (3)
O3—La—O6	61.97 (9)	C12—C13—H13A	118.9
O14—La—O6	97.88 (12)	C14—C13—H13A	118.9
O5'—La—O6	42.8 (5)	C13—C14—C9	118.9 (4)
O5—La—O6	48.1 (4)	C13—C14—H14A	120.6
O11—La—O6	120.64 (11)	C9—C14—H14A	120.6
O8—La—O6	63.33 (12)	C12—C15—H15A	109.5
O12—La—O6	80.39 (10)	C12—C15—H15B	109.5
O9—La—O6	98.05 (11)	H15A—C15—H15B	109.5
O1—La—O4	74.24 (7)	C12—C15—H15C	109.5
O3—La—O4	59.36 (7)	H15A—C15—H15C	109.5
O14—La—O4	73.01 (12)	H15B—C15—H15C	109.5
O5'—La—O4	135.7 (4)	C22—C16—C21	119.2 (3)
O5—La—O4	151.3 (4)	C22—C16—C17	120.8 (3)
O11—La—O4	71.17 (9)	C21—C16—C17	120.0 (3)
O8—La—O4	134.08 (10)	O3—C17—C16	121.9 (3)
O12—La—O4	69.48 (9)	O3—C17—C18	121.1 (3)
O9—La—O4	136.83 (9)	C16—C17—C18	117.1 (3)
O6—La—O4	120.37 (9)	C19—C18—O4	125.7 (3)
O1—La—O2	56.74 (7)	C19—C18—C17	121.4 (3)
O3—La—O2	131.40 (8)	O4—C18—C17	113.0 (3)
O14—La—O2	62.19 (10)	C18—C19—C20	120.6 (3)
O5'—La—O2	68.6 (5)	C18—C19—H19A	119.7
O5—La—O2	63.1 (4)	C20—C19—H19A	119.7
O11—La—O2	114.18 (7)	C21—C20—C19	120.9 (3)
O8—La—O2	102.91 (9)	C21—C20—H20A	119.6
O12—La—O2	160.04 (8)	C19—C20—H20A	119.6
O9—La—O2	61.45 (9)	C20—C21—C16	120.1 (3)
O6—La—O2	110.58 (9)	C20—C21—H21A	120.0
O4—La—O2	115.03 (8)	C16—C21—H21A	120.0
C2—O1—La	133.08 (19)	N2—C22—C16	124.7 (3)
C3—O2—C8	115.9 (4)	N2—C22—H22A	117.7
C3—O2—La	114.91 (19)	C16—C22—H22A	117.7
C8—O2—La	128.8 (3)	O4—C23—H23A	109.5
C17—O3—La	128.9 (2)	O4—C23—H23B	109.5
C18—O4—C23	117.0 (3)	H23A—C23—H23B	109.5
C18—O4—La	117.00 (18)	O4—C23—H23C	109.5
C23—O4—La	125.3 (3)	H23A—C23—H23C	109.5
N3—O5—La	97.9 (10)	H23B—C23—H23C	109.5
N3—O5'—La	105.2 (13)	C25—C24—C29	118.7 (4)
N3—O6—La	94.6 (2)	C25—C24—N2	118.8 (3)
N4—O8—La	98.3 (2)	C29—C24—N2	122.5 (3)
N4—O9—La	97.8 (2)	C24—C25—C26	120.6 (4)
N5—O11—La	98.73 (19)	C24—C25—H25A	119.7
N5—O12—La	96.91 (18)	C26—C25—H25A	119.7
C31—O14—La	131.9 (3)	C27—C26—C25	121.9 (4)
C31—O14—H14	112 (4)	C27—C26—H26A	119.0
La—O14—H14	116 (4)	C25—C26—H26A	119.0
C7—N1—C9	126.5 (3)	C26—C27—C28	116.8 (4)
C7—N1—H1A	116.7	C26—C27—C30	121.1 (5)
C9—N1—H1A	116.7	C28—C27—C30	122.1 (5)
C22—N2—C24	127.3 (3)	C29—C28—C27	122.4 (5)
C22—N2—H2A	116.4	C29—C28—H28A	118.8
C24—N2—H2A	116.4	C27—C28—H28A	118.8
O5'—N3—O6	112.0 (11)	C24—C29—C28	119.7 (4)
O5'—N3—O7	123.3 (11)	C24—C29—H29A	120.2
O6—N3—O7	122.0 (4)	C28—C29—H29A	120.2
O5'—N3—O5	32.1 (13)	C27—C30—H30A	109.5
O6—N3—O5	117.7 (9)	C27—C30—H30B	109.5
O7—N3—O5	118.3 (9)	H30A—C30—H30B	109.5
O9—N4—O10	121.4 (3)	C27—C30—H30C	109.5
O9—N4—O8	117.3 (3)	H30A—C30—H30C	109.5
O10—N4—O8	121.3 (3)	H30B—C30—H30C	109.5
O13—N5—O11	121.3 (3)	O14—C31—H31A	109.5
O13—N5—O12	121.6 (3)	O14—C31—H31B	109.5
O11—N5—O12	117.1 (3)	H31A—C31—H31B	109.5
C7—C1—C2	121.5 (3)	O14—C31—H31C	109.5
C7—C1—C6	118.1 (3)	H31A—C31—H31C	109.5
C2—C1—C6	120.4 (3)	H31B—C31—H31C	109.5
			
O3—La—O1—C2	−131.3 (3)	O1—La—O11—N5	161.2 (2)
O14—La—O1—C2	−74.0 (3)	O3—La—O11—N5	33.3 (2)
O5'—La—O1—C2	−12.8 (6)	O14—La—O11—N5	115.4 (2)
O5—La—O1—C2	3.6 (5)	O5'—La—O11—N5	−80.9 (10)
O11—La—O1—C2	135.5 (3)	O5—La—O11—N5	−94.4 (6)
O8—La—O1—C2	80.0 (3)	O8—La—O11—N5	−74.5 (2)
O12—La—O1—C2	150.9 (3)	O12—La—O11—N5	1.05 (18)
O9—La—O1—C2	55.7 (3)	O9—La—O11—N5	−123.8 (2)
O6—La—O1—C2	21.1 (5)	O6—La—O11—N5	−34.6 (2)
O4—La—O1—C2	−148.2 (3)	O4—La—O11—N5	80.1 (2)
O2—La—O1—C2	−12.9 (3)	O2—La—O11—N5	−170.17 (19)
O1—La—O2—C3	9.7 (2)	O1—La—O12—N5	−20.1 (2)
O3—La—O2—C3	128.2 (2)	O3—La—O12—N5	−148.1 (2)
O14—La—O2—C3	110.3 (3)	O14—La—O12—N5	−120.0 (2)
O5'—La—O2—C3	−170.3 (5)	O5'—La—O12—N5	144.1 (5)
O5—La—O2—C3	−154.0 (5)	O5—La—O12—N5	125.2 (4)
O11—La—O2—C3	−21.6 (3)	O11—La—O12—N5	−1.04 (18)
O8—La—O2—C3	−95.5 (2)	O8—La—O12—N5	83.4 (2)
O12—La—O2—C3	−40.7 (4)	O9—La—O12—N5	52.0 (2)
O9—La—O2—C3	−73.5 (2)	O6—La—O12—N5	148.4 (2)
O6—La—O2—C3	−161.5 (2)	O4—La—O12—N5	−84.0 (2)
O4—La—O2—C3	58.0 (3)	O2—La—O12—N5	23.0 (4)
O1—La—O2—C8	−177.5 (6)	O1—La—O14—C31	−171.4 (6)
O3—La—O2—C8	−58.9 (6)	O3—La—O14—C31	−33.1 (6)
O14—La—O2—C8	−76.8 (6)	O5'—La—O14—C31	57.6 (8)
O5'—La—O2—C8	2.6 (7)	O5—La—O14—C31	69.3 (7)
O5—La—O2—C8	18.9 (7)	O11—La—O14—C31	−130.7 (6)
O11—La—O2—C8	151.3 (6)	O8—La—O14—C31	69.3 (7)
O8—La—O2—C8	77.4 (6)	O12—La—O14—C31	−60.6 (7)
O12—La—O2—C8	132.1 (6)	O9—La—O14—C31	128.8 (6)
O9—La—O2—C8	99.4 (6)	O6—La—O14—C31	23.6 (6)
O6—La—O2—C8	11.3 (6)	O4—La—O14—C31	−95.8 (6)
O4—La—O2—C8	−129.2 (6)	O2—La—O14—C31	132.8 (6)
O1—La—O3—C17	−25.9 (3)	La—O5'—N3—O6	24.1 (11)
O14—La—O3—C17	−87.9 (3)	La—O5'—N3—O7	−174.5 (4)
O5'—La—O3—C17	−159.9 (5)	La—O5'—N3—O5	−83 (3)
O5—La—O3—C17	−166.5 (5)	La—O6—N3—O5'	−21.8 (10)
O11—La—O3—C17	46.5 (3)	La—O6—N3—O7	176.6 (4)
O8—La—O3—C17	122.2 (3)	La—O6—N3—O5	13.1 (9)
O12—La—O3—C17	71.4 (3)	La—O5—N3—O5'	74 (3)
O9—La—O3—C17	136.3 (3)	La—O5—N3—O6	−13.9 (10)
O6—La—O3—C17	161.9 (3)	La—O5—N3—O7	−178.0 (4)
O4—La—O3—C17	−6.9 (2)	La—O9—N4—O10	179.3 (3)
O2—La—O3—C17	−104.6 (3)	La—O9—N4—O8	−1.8 (4)
O1—La—O4—C18	172.4 (3)	La—O8—N4—O9	1.9 (4)
O3—La—O4—C18	7.0 (2)	La—O8—N4—O10	−179.3 (3)
O14—La—O4—C18	84.9 (2)	La—O11—N5—O13	178.8 (3)
O5'—La—O4—C18	47.5 (7)	La—O11—N5—O12	−1.8 (3)
O5—La—O4—C18	52.8 (9)	La—O12—N5—O13	−178.9 (3)
O11—La—O4—C18	−119.5 (2)	La—O12—N5—O11	1.8 (3)
O8—La—O4—C18	−85.4 (3)	La—O1—C2—C3	14.8 (4)
O12—La—O4—C18	−69.2 (2)	La—O1—C2—C1	−165.3 (2)
O9—La—O4—C18	−153.9 (2)	C7—C1—C2—O1	3.6 (5)
O6—La—O4—C18	−4.4 (3)	C6—C1—C2—O1	−176.7 (4)
O2—La—O4—C18	131.9 (2)	C7—C1—C2—C3	−176.5 (3)
O1—La—O4—C23	2.6 (4)	C6—C1—C2—C3	3.2 (5)
O3—La—O4—C23	−162.7 (4)	C8—O2—C3—C4	−0.7 (7)
O14—La—O4—C23	−84.9 (4)	La—O2—C3—C4	173.1 (3)
O5'—La—O4—C23	−122.3 (8)	C8—O2—C3—C2	178.7 (5)
O5—La—O4—C23	−117.0 (9)	La—O2—C3—C2	−7.5 (4)
O11—La—O4—C23	70.7 (4)	O1—C2—C3—O2	−1.7 (4)
O8—La—O4—C23	104.8 (4)	C1—C2—C3—O2	178.4 (3)
O12—La—O4—C23	121.0 (4)	O1—C2—C3—C4	177.8 (3)
O9—La—O4—C23	36.3 (4)	C1—C2—C3—C4	−2.2 (5)
O6—La—O4—C23	−174.2 (4)	O2—C3—C4—C5	180.0 (4)
O2—La—O4—C23	−37.9 (4)	C2—C3—C4—C5	0.6 (6)
O1—La—O5—N3	−178.7 (6)	C3—C4—C5—C6	−0.1 (8)
O3—La—O5—N3	−31.2 (9)	C4—C5—C6—C1	1.2 (8)
O14—La—O5—N3	−100.7 (8)	C7—C1—C6—C5	176.9 (5)
O5'—La—O5—N3	−56 (3)	C2—C1—C6—C5	−2.8 (7)
O11—La—O5—N3	99.4 (8)	C9—N1—C7—C1	−178.2 (4)
O8—La—O5—N3	79.4 (8)	C2—C1—C7—N1	7.1 (6)
O12—La—O5—N3	38.7 (10)	C6—C1—C7—N1	−172.6 (4)
O9—La—O5—N3	129.3 (9)	C7—N1—C9—C10	−168.9 (4)
O6—La—O5—N3	7.2 (5)	C7—N1—C9—C14	11.1 (6)
O4—La—O5—N3	−69.8 (13)	C14—C9—C10—C11	1.5 (6)
O2—La—O5—N3	−163.3 (10)	N1—C9—C10—C11	−178.6 (4)
O1—La—O5'—N3	153.7 (9)	C9—C10—C11—C12	−0.3 (7)
O3—La—O5'—N3	−67.7 (11)	C10—C11—C12—C13	−1.5 (7)
O14—La—O5'—N3	−139.6 (12)	C10—C11—C12—C15	177.6 (5)
O5—La—O5'—N3	88 (3)	C11—C12—C13—C14	2.2 (7)
O11—La—O5'—N3	52.1 (17)	C15—C12—C13—C14	−176.9 (5)
O8—La—O5'—N3	46.0 (10)	C12—C13—C14—C9	−1.1 (7)
O12—La—O5'—N3	−7.9 (13)	C10—C9—C14—C13	−0.8 (6)
O9—La—O5'—N3	92.7 (11)	N1—C9—C14—C13	179.2 (4)
O6—La—O5'—N3	−14.2 (7)	La—O3—C17—C16	−174.5 (2)
O4—La—O5'—N3	−101.8 (11)	La—O3—C17—C18	6.2 (4)
O2—La—O5'—N3	153.8 (12)	C22—C16—C17—O3	−4.4 (5)
O1—La—O6—N3	−28.9 (6)	C21—C16—C17—O3	177.4 (3)
O3—La—O6—N3	127.9 (3)	C22—C16—C17—C18	175.0 (3)
O14—La—O6—N3	64.2 (3)	C21—C16—C17—C18	−3.2 (4)
O5'—La—O6—N3	13.1 (6)	C23—O4—C18—C19	−16.6 (6)
O5—La—O6—N3	−8.0 (5)	La—O4—C18—C19	172.7 (3)
O11—La—O6—N3	−135.9 (3)	C23—O4—C18—C17	163.4 (4)
O8—La—O6—N3	−93.5 (3)	La—O4—C18—C17	−7.2 (4)
O12—La—O6—N3	−161.7 (3)	O3—C17—C18—C19	−178.1 (3)
O9—La—O6—N3	−61.4 (3)	C16—C17—C18—C19	2.5 (5)
O4—La—O6—N3	139.1 (3)	O3—C17—C18—O4	1.8 (4)
O2—La—O6—N3	1.0 (3)	C16—C17—C18—O4	−177.6 (3)
O1—La—O8—N4	−33.1 (3)	O4—C18—C19—C20	179.8 (3)
O3—La—O8—N4	171.8 (2)	C17—C18—C19—C20	−0.3 (6)
O14—La—O8—N4	80.2 (3)	C18—C19—C20—C21	−1.2 (6)
O5'—La—O8—N4	91.4 (5)	C19—C20—C21—C16	0.4 (6)
O5—La—O8—N4	80.2 (5)	C22—C16—C21—C20	−176.3 (3)
O11—La—O8—N4	−85.5 (3)	C17—C16—C21—C20	1.9 (5)
O12—La—O8—N4	−136.4 (3)	C24—N2—C22—C16	−175.9 (3)
O9—La—O8—N4	−1.0 (2)	C21—C16—C22—N2	−179.3 (3)
O6—La—O8—N4	132.6 (3)	C17—C16—C22—N2	2.5 (5)
O4—La—O8—N4	−119.8 (2)	C22—N2—C24—C25	175.6 (4)
O2—La—O8—N4	25.9 (3)	C22—N2—C24—C29	−2.3 (6)
O1—La—O9—N4	148.8 (3)	C29—C24—C25—C26	−0.4 (7)
O3—La—O9—N4	−17.0 (4)	N2—C24—C25—C26	−178.4 (4)
O14—La—O9—N4	−144.7 (2)	C24—C25—C26—C27	0.5 (8)
O5'—La—O9—N4	−80.7 (5)	C25—C26—C27—C28	0.1 (8)
O5—La—O9—N4	−79.2 (5)	C25—C26—C27—C30	179.3 (5)
O11—La—O9—N4	80.0 (2)	C26—C27—C28—C29	−0.7 (9)
O8—La—O9—N4	1.1 (2)	C30—C27—C28—C29	−179.9 (6)
O12—La—O9—N4	42.1 (3)	C25—C24—C29—C28	−0.2 (8)
O6—La—O9—N4	−39.7 (3)	N2—C24—C29—C28	177.6 (5)
O4—La—O9—N4	114.1 (2)	C27—C28—C29—C24	0.8 (9)
O2—La—O9—N4	−148.8 (3)		

### Hydrogen-bond geometry (Å, °)

**Table:**

*D*—H···*A*	*D*—H	H···*A*	*D*···*A*	*D*—H···*A*
N1—H1A···O1	0.86	2.01	2.668 (3)	132
N1—H1A···O11	0.86	2.52	3.307 (4)	153
N2—H2A···O3	0.86	1.95	2.627 (3)	135
N2—H2A···O6	0.86	2.65	3.440 (5)	154
